# Supplementary material for: Allocating limited surveillance effort for outbreak detection of endemic foot and mouth disease
Source: PLoS Comput Biol. 2025 Jul 11;21(7):e1012395. doi: 10.1371/journal.pcbi.1012395 (PMC12273912; doi:10.1371/journal.pcbi.1012395)
Supplement: Attachment — Submitted filename: PLOS review.docx [file pcbi.1012395.s003.docx]

PLOS review

The paper is interesting and well-written

Some general comments:

As there are potential disincentives for passive surveillance (farm restrictions, culling of animals etc following disease detection) that also pertain to the proposed sentinel surveillance, this should be raised somewhere in the text. In addition, the probability of animal movement data being accessible in low-resource settings should be touched upon. These aspects are important for the generalisability of the study.

Other options for targeted surveillance could also be discussed, such as focusing on markets and slaughterhouses and other hubs that receive animals from many farms. Surveillance in the data means passive clinical surveillance (i.e. farmers calling veterinarians or officials when their animals show symptoms), if you mean something else (e.g. sending animal health staff to farms for clinical check or sampling) this should be clarified and discussed. It does make a difference if the surveillance entails farm visits, and also if it relies on clinical signs or clinical signs plus sampling (e.g. for virus before signs appear).

Detailed comments:

L 100: I suggest replacing ‘fewer cattle deaths’ by ‘fewer cattle culled’ to clarify that they did not die from FMD

L 105: As the reference cited is rather old and focuses on public health and not animal health, I suggest removing it or replacing it by a textbook or the WOAH terrestrial code chapter on animal health surveillance. For the practising veterinarian, it may be somewhat difficult to see why this sentinel surveillance system would work better than asking all farmers to report outbreaks to their vet (which is the case in most legislation globally). It would be helpful to clarify how better compliance would be achieved in sentinel farms.

L 130: I suggest adding ‘passive’ (…extensive passive surveillance system to help…) for clarification

L138: This sentence is slightly confusing, I suggest rephrasing to ‘…demonstrating the usefulness of network analysis for assessing the persistence of FMD and for identifying high risk epiunits’

L146-147: it would be helpful with some examples of countries/regions where the implementation of the results could be foreseen. As minimal surveillance infrastructure often means that movement data are lacking, some examples where this is not the case would help argue the usefulness of the study

L 180-181: I suggest rephrasing to ‘If herds were confirmed positive, viral strain typing was usually performed.’ or similar, as the sentence is now slightly confusing.

L208-209: I suggest replacing this reference with the original WOAH reference that is cited in the document. In addition, the term serial interval does not seem to appar in the document and requires further explanation. It would logically not take up to 20 d for one cor to be able to transmit FMD after infection (as it starts shedding before showing clinical signs) so the explanation needs to be rephrased, perhaps you mean the interval between infecting one animal and a secondary case showing clinical signs after being infected by the first one?

L286: Please add ‘to’ (sent to or received cattle from)

L517-523: It is not entirely surprising that serotype is not important here, the detection method in the data is based on clinical signs. Perhaps comment on this.

L527-528: It would be useful to discuss the All-Month Network Connectivity vs the Two-Months in a context with stable movement networks vs fluctuating (i.e. if high-risk herds more constant or always changing), as the network characteristics might influence the results.

L534-537: Doesn’t the usefulness of different prioritisations depend on the purpose of surveillance, early detection or outbreak tracing? If early detection is aimed for, then you cannot wait for outbreak data to guide you.

L549: More resources would assume that legislation and registers are available, this should be clarified.

L606: Were there any trends in the movement data suggesting that movement restrictions were in place (and worked) around outbreaks?

In figure 2 and 3 it would be useful to also include the All-Month Network Connectivity as this is what might be used preventively. In addition, the 2-month network measure assumes a continuous re-prioritisation in farms surveyed.

L626-627 Could you expand slightly on the dynamic model, do you mean based on this dataset or wouldn’t you need more examples to test this?

L636-637: It would be helpful with a slight expansion on what the increase in herd coverage would mean in practice, would it entail more resources being allocated (animal health staff for clinical surveillance or sampling?).
